# Approval of Raxibacumab for the Treatment of Inhalation Anthrax Under the US Food and Drug Administration “Animal Rule”

**DOI:** 10.3389/fmicb.2015.01320

**Published:** 2015-12-01

**Authors:** Chia-Wei Tsai, Stephen Morris

**Affiliations:** ^1^Biomedical Advanced Research and Development Authority, Office of the Assistant Secretary for Preparedness and Response, U.S. Department of Health and Human ServicesWashington, DC, USA; ^2^BioProtection Systems/NewLink Genetics Corp.Devens, MA, USA

**Keywords:** Raxibacumab, animal rule, FDA licensure, anthrax therapeutics, anthropometry

## Abstract

On December 14, 2012, the FDA approved Raxibacumab, the first monoclonal antibody product developed under Project BioShield to achieve this milestone, and the first biologic product to be approved through the FDA animal efficacy rule (or “Animal Rule”). Raxibacumab is approved for the treatment of adult and pediatric patients with inhalational anthrax due to *Bacillus anthracis* in combination with appropriate antibiotic drugs and for prophylaxis of inhalational anthrax when alternative therapies are not available or not appropriate. The developmental process required for approval of Raxibacumab illustrates many of the challenges that product developers may encounter when pursuing approval under the Animal Rule and highlights a number of important regulatory and policy issues.

## The establishment of anthrax response capabilities for US government

In 2004 Congress passed the Project Bioshield Act providing the organizational infrastructure and money to acquire medical countermeasures for civilian use in the event of a bioterrorist attack. Medical countermeasure development and procurement is managed by an interagency body known as the Public Health Emergency Medical Countermeasures Enterprise (PHEMCE), including the Biomedical Advanced Research and Development Authority (BARDA) responsible for the late-stage development of medical countermeasures. The priorities for countermeasure development are established in accordance with provisions of the Project BioShield Act of 2004 with the Department of Homeland Security (DHS) determining which agents present “a material threat against the United States population sufficient to affect national security.” The establishment of a material threat is required for the Department of Health and Human Services (HHS) to pursue countermeasure development using funds available under Project BioShield for the development and procurement of medical countermeasures for the U.S. Strategic National Stockpile (SNS). Anthrax, and in particular anthrax disease caused by inhalation of anthrax spores, was designated a material threat by DHS.

## Development of Raxibacumab for anthrax treatment

As a result of the anthrax attacks in 2001, 11 people developed inhalational anthrax, and despite intensive care including treatment with multiple antibiotics, five of them died. Although this mortality rate of 45% was lower than the 90% rate observed historically, the high mortality resulting from this attack suggests that antibiotic therapy alone is insufficient for the treatment of such patients (Inglesby et al., 2002; Holty et al., 2006). In a concerted attack with an outdoor release of anthrax modeling suggests that large numbers of people would be exposed with a substantial fraction developing symptomatic inhalational anthrax, even in the setting of a prompt public health response (Jernigan et al., 2001).

Antibiotics can be used to treat anthrax infection by reducing bacteremia. However, since antibiotics have no direct effect on toxins they cannot address the toxemia that is responsible for the majority of the morbidity and mortality associated with anthrax infection (Inglesby et al., 2002). In humans, as well as animals, inhalational anthrax disease occurs following inhalation of *Bacillus anthracis* spores, which germinate within macrophages as they travel to the draining mediastinal lymph nodes of the lung. Multiplication of the bacteria results in a high organism count in the blood, production of bacterial toxins, and the rapid onset of septicemia (Cote et al., 2011). Although the bacterial replication (bacteremia) can be controlled by administration of appropriate antibiotics, the bacterial toxin exerts deleterious effects on the cells within the body, resulting in substantial pathology and high mortality in infected individuals. If the toxin reaches sufficient levels in an individual, controlling bacterial replication with an antibiotic will not be sufficient to affect the clinical outcome of the patient (Chitlaru et al., 2011).

Medical treatments against anthrax, such as antibiotics, provide time for the host to mount an effective immune response to clear the bacteria. All survivors of the 2001 anthrax attacks developed an immune response to the anthrax toxin by day 28 after exposure (Wright et al., 2010). There is an FDA-licensed anthrax vaccine, Anthrax Vaccine Adsorbed (AVA) (http://www.fda.gov/BiologicsBloodVaccines/Vaccines/ApprovedProducts/ucm133822.htm), which works by inducing an immune response primarily to the protective antigen component of anthrax toxin. Once subjects produce this antibody, they are protected against the effects of anthrax. However, vaccination is not an effective stand-alone therapy after infection since anthrax disease progresses rapidly and is fatal long before the time required to generate an immune response from vaccination. Immunization against anthrax requires multiple doses of vaccine and more than 2 months to induce protective titers of antitoxin antibody (Wright et al., 2010).

Immediately after the anthrax attacks in September and October of 2001, Human Genome Sciences, Inc. (HGS) embarked on a development program to produce a monoclonal antibody to treat inhalational anthrax. The product of this effort, Raxibacumab, is a human recombinant monoclonal antibody that is administered by the intravenous route, resulting in the rapid delivery of a PA-targeted antitoxin to patients. At the approved dosage, Raxibacumab halts the deleterious effects of toxin, providing time to “bridge the gap” for patients until endogenous antibodies are produced and immunity acquired. Antitoxin therapy treats the toxemia component of anthrax infection that antibiotics cannot address (Migone et al., 2009; Raxibacumab for anthrax, 2013). The efficacy of Raxibacumab was demonstrated by its ability to improve survival after lethal aerosol challenges of anthrax in two animal models, New Zealand White rabbits and cynomologus macaque nonhuman primates. In both models, Raxibacumab treatment after exposure to aerosolized anthrax led to a higher percentage of survival compared to untreated animals when administered early in the disease, before symptoms develop, as well as later, when the disease has progressed to systemic infection (Mazumdar, 2009; Migone et al., 2009; Corey et al., 2013).

In December 2012, the United States Food and Drug Administration (FDA) approved Raxibacumab for the treatment of and prophylaxis against inhalational anthrax (http://www.fda.gov/NewsEvents/Newsroom/PressAnnouncements/ucm332341.htm). Also in 2012, HGS was acquired by GlaxoSmithKline (GSK), which assumed the product license and the responsibility for post marketing commitments and production of Raxibacumab. Raxibacumab was shown to be safe at the approved dose (40 mg/kg) through clinical studies in more than 300 healthy adults including 43 individuals receiving a double dose (Subramanian et al., 2005; Migone et al., 2009).

## Regulatory path under FDA's animal rule

In order to provide a regulatory path for the evaluation of medical countermeasures against biological threats the FDA published in 2002 a regulatory guideline, “Evidence Needed to Demonstrate Effectiveness of New Drugs When Human Efficacy Studies Are Not Ethical or Feasible,” which was updated in 2015 (21 CFR 601, Subpart H; Food and Drug Administration, 2002) (Animal Rule, 2015). This guideline, known as the “Animal Rule”, described a regulatory pathway that has been used to support the development of medical countermeasures against chemical, biological, radiological, or nuclear (CBRN) threats. Raxibacumab is the first new drug to receive approval under the Animal Rule.

The “Animal Rule” describes a process to determine the efficacy of a medical countermeasure, such as Raxibacumab, by performing pivotal trails in animals in lieu of clinical trials in humans. The studies must address the four criteria for demonstration of efficacy under the Animal Rule:

### The product has a well-understood mechanism of action against a target with known pathophysiology

The mechanism of anthrax toxin toxicity has been extensively studied *in vitro* and the role of protective antigen (PA) in anthrax disease is well-documented. *In vitro* studies demonstrate that Raxibacumab binds PA with high affinity and interferes with the binding of PA to its receptor thereby preventing the killing of murine and human macrophages by anthrax toxins (Mazumdar, 2009; Chen et al., 2011). Further *in vivo* studies in the rat, rabbit, and cynomolgus macaque monkey demonstrated that treatment with Raxibacumab provides protection from the lethal effects of anthrax toxins leading to survival of infected animals (Cui et al., 2005; Mazumdar, 2009; Migone et al., 2009; Corey et al., 2013).

### Demonstration of benefit in more than one animal species, unless a single species represents a sufficiently well-characterized model to predict response in humans

HGS demonstrated the efficacy of Raxibacumab in both the New Zealand White rabbit and cynomolgus macaque monkey anthrax inhalation models. Both models were considered to provide predictive value on the efficacy of anthrax treatments since in both animal models the contribution of bacteremia and toxemia to mortality were similar to that observed for humans. Pivotal efficacy studies were conducted in both models (Migone et al., 2009; Corey et al., 2013).

### Use of an efficacy endpoint relevant to the desired outcome in humans

The primary endpoint used in the animal studies, mortality, is relevant to the outcome of the human disease, which is highly lethal. In both rabbits and monkeys exposed to a target anthrax spore challenge of 200 × lethal dose (LD_50_) almost 100% mortality was observed. Treatment of exposed animals with Raxibacumab provided statistically significant improvement in survival rates and in those animals that died, an extended time to death.

In the pivotal rabbit efficacy studies, none of the animals in the control groups (rabbit or nonhuman primate) challenged with 200 LD_50_ aerosolized anthrax spores survived. When rabbits were challenged with 200 LD_50_ aerosolized anthrax spores and treated with Raxibacumab 44.4% of animals treated with 40 mg/kg survived (*p* = 0.0029). Likewise, in the pivotal study with nonhuman primates, 64.3% (*p* = 0.0007) of those treated with 40 mg/kg survived. These results and the details of the challenge model are presented in the cited reference (Migone et al., 2015).

One of the outcomes of the Vaccines and Related Biological Products Advisory Committee (VRBPAC) meeting on Raxibacumab was an FDA request to investigate the concomitant use of the antitoxin with antibiotics, the present standard of care for anthrax disease. The Agency wanted to determine if co-administration of antitoxin would affect the efficacy and pharmokinetic (PK) parameters of antibiotics and to determine if the addition of Raxibacumab to antibiotic treatment would provide an “added benefit” in increased efficacy. Co-administration of Raxibacumab did not alter the efficacy of levofloxacin (rabbits) or ciprofloxacin (nonhuman primates) administered at recommended human doses (Migone et al., 2009; Corey et al., 2013). Also, a clinical trial demonstrated that the PK parameters of ciprofloxacin were unaffected with concomitant use of Raxibacumab.

The demonstration of “added benefit” was more problematic since early administration of antibiotics resulted in nearly 100% survival in both the rabbit and nonhuman primate model, thus making it very difficult to demonstrate additional value with Raxibacumab use. To accomplish this, a “delayed treatment” model was developed in which antibiotic treatment was delayed so that the survival rate of exposed animals resembles the rate observed for humans during the 2001 anthrax attacks (~55% rather than the survival rate of close to 100% for animals treated when systemic anthrax disease was detected).

A delayed treatment study in the New Zealand White rabbit model was used to support regulatory evaluation of Raxibacumab. In this study treatment with the antibiotic levofloxacin was delayed until 84 h post-exposure at which time 42% of the animals remained alive. The remaining animals were randomized into two groups for treatment with levofloxacin or a combination of Raxibacumab and levofloxacin. The survival rate for animals treated with levofloxacin was 65% (24/37) compared to 82% (32/39) of the rabbits in the treatment group receiving levofloxacin and Raxibacumab. The difference in the survival did not reach the *p* = 0.05 level of statistical significance (*p* = 0.07) however the November 2, 2012 Anti-Infective Drugs Advisory Committee voted overwhelmingly that these results demonstrated the co-administration of Raxibacumab and antibiotics would provide clinical benefit.

### Pharmacokinetic data that allow translation of effective animal doses to recommended human doses

In order to be confident that the efficacy results in animal models are predictive of the countermeasure efficacy in humans the FDA requires demonstration that the exposure levels of the drug in humans meets or exceeds the exposure level that is effective in the animal model. The PK data for Raxibacumab have been acquired in both uninfected and anthrax infected rabbits and cynomolgus monkeys. The PK data for humans was determined from clinical trials including single and repeat dosing as well as co-administration with antibiotics. The PK parameters of the approved dose (40 mg/kg) in humans were acceptable for FDA approval.

## Safety

Although efficacy is evaluated through animal models under the FDA Animal Rule, safety is demonstrated through well-monitored clinical trials. Considering the indicated use of Raxibacumab as a therapeutic, the FDA required a safety database of over 300 healthy human volunteers before approval. Adverse events (AEs) were generally mild to moderate and did not occur at a rate that was different from that observed among placebo-treated subjects. A low incidence of mild to moderate rash was observed in some subjects. These rashes were transient and resolved without medication or with oral diphenhydramine. Concomitant administration of Raxibacumab with ciprofloxacin did not alter the safety or PK of either antibiotic or Raxibacumab (Subramanian et al., 2005).

## Post marketing requirement after the licensure

Based on the human safety data and the animal efficacy study results, the US FDA approved Raxibacumab under the Animal Rule for the treatment of adult and pediatric patients with inhalational anthrax due to *B. anthracis* in combination with appropriate antibiotic drugs, and for prophylaxis of inhalational anthrax when alternative therapies are not available or are not appropriate. Because efficacy data for product evaluation are collected from animal models, one of the requirements for products licensed under the Animal Rule is a post-marketing commitment from the product sponsor to collect efficacy and safety data post approval when the product is used in humans to treat anthrax. In anticipation of Raxibacumab deployment and use, GSK has submitted a field study protocol to the FAD to evaluate the effectiveness, pharmacokinetics and safety of Raxibacumab use for *B. anthracis* infection in the United States. This is an open-label, single arm, study to evaluate the clinical course (including survival), safety and PK in men, women, and children of all ages exposed to *B. anthracis* and treated with Raxibacumab as part of their clinical care (https://clinicaltrials.gov/ct2/show/NCT02177721). The protocol applies to circumstances where Raxibacumab is deployed to treat individual cases of anthrax as well as an emergency where mass distribution of Raxibacumab may be required. Once Raxibacumab is dispatched by CDC, the field study will be rapidly implemented to collect important clinical and PK data on Raxibacumab in the midst of a public health emergency. The study data will be used to confirm the efficacy and safety of Raxibacumab and further inform patient care and treatment choices for management of anthrax.

GSK also has a post-marketing commitment to evaluate the effect of concomitant administration of Raxibacumab on the activity of the licensed anthrax vaccine BioThrax® when used under conditions of post-exposure prophylaxis. In 2002, the CDC recommended use of BioThrax® in conjunction with antibiotics in the post-exposure-prophylaxis setting, with administration of the vaccine at 0, 2, and 4 weeks post exposure (Wright et al., 2010). The FDA was concerned with the ability of the vaccine to elicit a protective immune response in a scenario where antitoxin and vaccine were co-administered to individuals during an anthrax emergency. Since the primary antigen in Biothrax® is PA, it is possible that concurrent administration of BioThrax® and Raxibacumab would result in Raxibacumab binding the PA from BioThrax®, leading to a reduced immune response to the vaccine.

To satisfy this post-marketing commitment GSK is executing a clinical study designed to detect the effect of Raxibacumab on Biothrax® immunogenicity in a healthy volunteer population (https://clinicaltrials.gov/ct2/show/NCT02339155).

## Challenges for Raxibacumab approval

The approval of Raxibacumab represents the first time a monoclonal antibody product was evaluated for efficacy under the FDA Animal Rule. The low number of inhalational anthrax cases made it impossible to demonstrate efficacy using traditional regulatory pathways and human clinical trials. Therefore, the FDA is forced to estimate effectiveness of a product by “bridging” from efficacy results obtained in animal models. The need to build a convincing body of data demonstrating efficacy is more complicated than the traditional method of demonstrating efficacy through clinical trials or epidemiological results. The lack of clear clinical efficacy data forces the development pathway into an iterative process with significant lead time in animal model development and characterization prior to proof of concept or pivotal efficacy studies. The inability to perform clinical trials usually means that additional experiments are required to address uncertainties that arise during the development pathway. For example, the results from initial animal efficacy experiments led to reviewers' questions that required additional numerous studies to establish the “delayed treatment” model to investigate added benefit and a series of studies with intensive pathological characterization to determine the cause of the higher incidence of brain lesions in animals that died after treatment with Raxibacumab. These studies added 2 years and over $10 million dollars to the developmental pathway. Also, the lack of clinical evidence for efficacy means there are significant post-marketing commitments to ensure data collection in the event the products are used in humans during an anthrax emergency.

It is difficult to compare the cost and time for product development under the Animal Rule to that using the traditional pathways relying on human clinical trials. The studies needed to support early development of Raxibacumab were supported by Human Genome Science internal resources before transition to BARDA in 2007. Support from NIH was also instrumental in the characterization of the aerosol challenge animal model and in the development of critical assays for the pivotal trials. Using a rough estimate it appears that the time required for product development and the cost associated with this development is not vastly different from that of a product using traditional regulatory pathways. The ability to develop medical countermeasures using the Animal Rule, however, provides an enormous benefit to the public health community in preparation for a biological attack. In the absence of the Animal Rule, anthrax countermeasures would be under IND status, making the deployment and use of countermeasures difficult in a public health emergency.

## Conclusion

Since the approval of Raxibacumab, two other medical countermeasures supported by BARDA, heptavalent Botulism Antitoxin (hBAT), and Anthrax Immune Globulin (AIG), were also approved, and three other medical countermeasures are in late stage development for evaluation using the Animal Rule. The experience gained through the Raxibacumab approval was beneficial in the design of approaches for the evaluation of the other products under the Animal Rule. In 2014 the FDA published a draft guidance entitled *Product Development Under the Animal Rule* to replace the version published in 2009. This revision covers the issues for product development under the Animal Rule including new sections on study conduct, data quality, data integrity, vaccine development, the development of cellular and gene therapies, general expectations for animal studies, the types of animals used in investigations, animal care, study reports, and natural history studies.

As product developers and the FDA continue to use the Animal Rule as a mechanism for efficacy evaluation, its role will continue to evolve. The most important lesson BARDA learned from the approval of Raxibacumab is that it is essential to engage the FDA review division early and often throughout the drug development process. Since evaluation of efficacy under the Animal Rule is an iterative process requiring a substantial body of data the best means of avoiding delays is frequent and open communication between sponsors and FDA. Although there are significant challenges associated with the evaluation of products using the Animal Rule since each product development process will present unique problems determined by the type of threat agent, the availability of well-characterized animal models, and the clarifying studies required during product evaluation this regulatory mechanism allows the USG to be prepared to deploy in an emergency products with a high likelihood of efficacy that have been evaluated using the highest standards for safety and manufacturing using good manufacturing practices as ensured by the FDA.

### Conflict of interest statement

The authors declare that the research was conducted in the absence of any commercial or financial relationships that could be construed as a potential conflict of interest.
